# A Collaborative Occupational Therapy–Instructor Model for Driving Evaluation of Persons with Disabilities

**DOI:** 10.3390/ijerph23050566

**Published:** 2026-04-27

**Authors:** Seongwon Kim, Seunghui Nina Jeong, Minye Jung, Yunjeong Eom, Jungran Kim, Meng-en Yang, Junghun Aj Kim

**Affiliations:** 1 Department of Occupational Therapy, Graduate School, Yonsei University, Wonju-si 26493, Gangwon-do, Republic of Korea; kswon94@yonsei.ac.kr (S.K.); uhmazing@yonsei.ac.kr (Y.E.); yme131543@yonsei.ac.kr (M.-e.Y.); 2 Department of Occupational Therapy, College of Software and Digital Healthcare Convergence, Yonsei University, Wonju 26493, Gangwon-do, Republic of Korea; minye@yonsei.ac.kr; 3 Department of Occupational Therapy, Dongnam Health University, Suwon-si 16327, Gyeonggi-do, Republic of Korea; rebirth79@dongnam.ac.kr; 4 Department of Occupational Therapy, Kangwon National University, Samcheok-si 25949, Gangwon-do, Republic of Korea

**Keywords:** driving rehabilitation, driver evaluation, persons with disabilities

## Abstract

**Highlights:**

**Public health relevance—How does this work relate to a public health issue?**

- Driving is essential for independence and community participation among persons with disabilities, yet standardized evaluation systems remain underdeveloped in Korea.
- Current practice relies on non-standardized, experience-based judgments, leading to inconsistent fitness-to-drive decisions.

**Public health significance—Why is this work of significance to public health?**

- This study developed and field-tested a structured comprehensive driving evaluation integrating off-road functional assessment with on-road driving performance.
- A collaborative model between occupational therapists and driving instructors improves the accuracy and consistency of driving fitness evaluation.

**Public health implications—What are the key implications or messages for practitioners, policy makers and/or researchers in public health?**

- The findings provide foundational evidence for establishing standardized driver evaluation systems for persons with disabilities in Korea.
- The proposed model supports policy development, interprofessional practice, and improved mobility services for persons with disabilities.

**Abstract:**

(1) Background: Driving enables participation and independence for persons with disabilities; however, Korea lacks standardized driver rehabilitation guidelines and clearly defined occupational therapy roles. Current evaluations at the National Rehabilitation Center (NRC) rely heavily on instructors’ experiential judgment, resulting in inconsistent fitness-to-drive decisions. This study developed and field-tested a comprehensive driving evaluation (CDE) tailored to the Korean service context, integrating structured off-road functional assessment with on-road driving evaluation through a collaborative occupational therapist–driving instructor model. (2) Methods: The off-road assessment was refined through a literature review, an analysis of the ICF Core Set for driving rehabilitation, expert surveys, and a workshop with 10 occupational therapists. The on-road assessment was adapted from international tools and validated by NRC driving instructors and an expert committee. The CDE was field-tested with 30 persons with physical disabilities, cerebral palsy, or auditory disabilities enrolled in the NRC Driving Education Program. Eligibility for independent driving was classified as “eligible” or “doubtful.” (3) Results: The CDE was feasible within existing workflows. Off-road deficits predicted on-road difficulties, and cases with discordant judgments benefited from combined assessment. (4) Conclusions: The CDE offers a structured alternative to experience-based evaluations and supports interprofessional collaboration, providing foundational evidence for standardized driver rehabilitation in Korea.

## 1. Introduction

Driving is a critical enabler of independence, community participation, and quality of life, particularly for persons with disabilities. However, assessing fitness to drive in this population requires specialized expertise and a structured, multidimensional evaluation process, as no single assessment tool has been found sufficient to predict driving safety [1,2,3]. Given the multidimensional nature of this field, related terms are often used interchangeably in the literature. In this paper, fitness to drive refers to the clinical determination of safe driving capacity [4]; driving evaluation refers to the structured assessment process used to inform such decisions [5]; and driver rehabilitation encompasses the broader service system including evaluation, intervention, and vehicle adaptation [1]. The present study focuses on developing and field-testing a comprehensive driving evaluation (CDE) that integrates off-road clinical assessment with on-road driving evaluation within a collaborative occupational therapist–driving instructor model.

Internationally, driver rehabilitation has evolved into a formalized area of practice in which occupational therapists play a central role. Surveys of driver rehabilitation specialists in North America have documented the assessment tools and procedures used in comprehensive driving evaluations [6,7], and competency standards for occupational therapy driver assessors have been developed and revised in Australia [3].

Several tools have been developed internationally to support fitness-to-drive assessment. In Australia, the Occupational Therapy–Driver Off-Road Assessment Battery (OT-DORA) provides a structured battery of clinical tests covering visual, cognitive, and motor domains [8]. In Sweden, the Performance Analysis of Driving Ability (P-Drive) enables observational scoring of on-road driving performance [9], and in Belgium, the Test Ride for Investigating Practical Fitness to Drive (TRIP) has been used as a standardized on-road assessment [10]. These tools reflect the growing international effort to move beyond informal judgment toward structured, evidence-informed evaluation procedures [6].

In Korea, by contrast, driving rehabilitation remains at an early stage of system development. Despite the existence of a national driving education program for persons with disabilities the concept of “driving rehabilitation” at the National Rehabilitation Center has not been formally defined in either healthcare or transportation legislation [11]. A national assessment guideline has not yet been fully established. As identified in prior work establishing an ICF Core Set for driving rehabilitation, service delivery has been fragmented, largely dependent on individual institutions and practitioners rather than a coordinated national model [12]. Historically, fitness-to-drive decisions at the National Rehabilitation Center (NRC) have relied primarily on the experiential judgment of a small number of instructors, with minimal functional criteria, limited interprofessional involvement, and only partial functional screening—most notably, the selective introduction of a single-item C-PAD test since 2020. This environment has hindered consistent practice, transparency, and equitable service access across the country [11,13].

In recent years, South Korea has undertaken legal and policy initiatives to enhance the mobility rights of persons with disabilities [14]. In parallel, national rehabilitation strategies have emphasized community reintegration and underscored the need for structured assessment pathways, workforce training, and standardized service protocols [13]. Occupational therapists, who internationally play a key role in clinical driver assessment, have only recently begun to be recognized as contributors to this service domain in Korea [15]. Integrating occupational therapy expertise with practical on-road instruction has been identified as essential for developing a safe and evidence-based driver rehabilitation system [16,17,18].

Integrating occupational therapy expertise with on-road driving evaluation has been widely recognized as a core component of comprehensive fitness-to-drive assessment [18,19]. In established systems, occupational therapists conduct off-road clinical assessments and collaborate with qualified driving instructors during behind-the-wheel evaluations to ensure both functional capacity and practical driving competence are considered [5].

To address these needs, our research team developed and validated a CDE tailored to persons with disabilities in Korea, building on the Screening Tool for Assessing Driving Ability [20] and the analysis of the ICF Core Set for driving rehabilitation [12]. The CDE incorporates standardized off-road assessments of physical, sensory, and cognitive functions, and evaluates real-world on-road performance.

Building on this foundation, the present study employs a collaborative model in which occupational therapists and driving instructors jointly evaluate and interpret assessment findings. Through a structured methodology involving expert review, stepwise refinement, and field implementation, this study aims to establish an objective, feasible, and clinically relevant approach to driving assessment that can serve as foundational evidence for national policy development, professional training, and the establishment of a coordinated driving rehabilitation system in Korea.

## 2. Materials and Methods

### 2.1. Study Design

The study was conducted in three phases. In Phase 1, the off-road driving assessment of the comprehensive driving evaluation for the present study was developed by revising and supplementing an assessment reported in a previous study [20]. In Phase 2, the on-road driving assessment of the comprehensive driving evaluation was developed through a literature review, preliminary drafting, and survey-based expert consultation with occupational therapists and driving instructors. In Phase 3, the comprehensive driving evaluation was field-tested within the NRC’s driving education program for persons with disabilities (Table 1).

### 2.2. Development of the Assessment Tools

#### 2.2.1. Development of the Off-Road Driving Assessment of the Comprehensive Driving Evaluation

The off-road driving assessment of the CDE was developed for this study by revising and supplementing the assessment reported by previous study [20]. The revision was conducted based on the opinions of an expert advisory committee composed of 10 occupational therapists working in rehabilitation hospitals, assistive device centers, and driver’s license support centers for persons with disabilities, all of whom had 4–21 years of clinical experience in driving rehabilitation. The committee members were selected based on their clinical experience in driving rehabilitation and their professional affiliations. Expert consultation was conducted through a structured survey and an online discussion. The collected data were analyzed using item-level descriptive statistics and content analysis. Based on this process, redundant items were removed, wording was refined, and context-specific elements relevant to Korean driving conditions and regulations were incorporated. The revised version was then used as the off-road driving assessment of the CDE in this study.

#### 2.2.2. Development of the On-Road Driving Assessment of the Comprehensive Driving Evaluation

The on-road driving assessment of the CDE was developed to assess the actual driving performance of persons with disabilities. The initial draft of the assessment items was constructed based on internationally recognized On-Road evaluation tools, including the Washington University Road Test (WURT) [21], the Performance Analysis of Driving Ability (P-Drive) [22], the Test Ride for Investigating Practical Fitness to Drive (TRIP) [23], and the University of Western Ontario On-Road Assessment (UWO On-Road) [24], as well as domestic road driving evaluation criteria.

To finalize the assessment items and the on-road evaluation form, a two-stage expert consultation process was conducted. In the first stage, a focus group interview (FGI) was conducted with seven driving instructors affiliated with the National Rehabilitation Center, a public institution that provides driving education for persons with disabilities, all of whom had 5 to 13 years of experience in this field. Participants were presented with the preliminary version of the on-road driving assessment form and were encouraged to freely discuss potential revisions and improvements. Their feedback regarding the feasibility, classification, and evaluation criteria of the assessment items was collected and reviewed.

In the second stage, a structured survey and an online discussion were conducted with ten occupational therapists who were members of the expert advisory committee and had previously participated in revising the off-road driving assessment. The collected data were analyzed using descriptive statistics and content analysis. Based on the findings, the assessment items were further refined and finalized, resulting in the completion of the on-road driving assessment of the CDE.

#### 2.2.3. Field Implementation of the Comprehensive Driving Evaluation

To examine the feasibility of real-world implementation of the developed assessment, the CDE was field-tested through the “Driving Education Program for persons with disabilities” organized by the NRC. The study recruited 30 persons with disabilities who expressed a desire to drive independently, including those classified under Korea’s disability categories of physical disability, brain lesions, and hearing impairment. After preliminary consultations to coordinate schedules and residential locations, each participant underwent both the off-road and on-road driving assessments. These assessments were jointly conducted by an occupational therapist and a driving instructor.

The occupational therapist first administered the off-road driving assessment to evaluate the participant’s physical, sensory, and cognitive functions and to identify considerations for on-road driving. These findings were communicated to the driving instructor in advance to ensure awareness of key safety issues during the on-road evaluation. During the on-road driving assessment, the driving instructor served as the primary evaluator, focusing on vehicle operation, directional control, and responses to traffic situations, while the occupational therapist sat in the back seat to observe the same driving situations and participate in the evaluation.

After completing the assessments, the occupational therapist and the driving instructor independently recorded their ratings and determined each participant’s fitness for independent driving. The two evaluators then compared their judgments, and any discrepancies were resolved through discussion based on observed driving performance and the results of each assessment. Final decisions were reached by consensus between the two evaluators. Based on the agreed findings, a final CDE report was prepared by integrating the physical, sensory, cognitive, and on-road driving results and classifying each participant as either ‘eligible’ or ‘doubtful’ for independent driving.

Data derived from the final CDE reports, including participants’ general characteristics and driving eligibility, were analyzed descriptively. General characteristics were summarized using frequencies and percentages, and the final classification results were summarized based on the integrated evaluation findings.

### 2.3. Ethical Considerations

This study was approved by the Institutional Review Board (IRB No. NRC-2024-03-021). All participants were fully informed of the study purpose and procedures and provided written consent prior to participation. All collected data were anonymized and used solely for research purposes.

## 3. Results

### 3.1. Development of the Comprehensive Driving Evaluation

#### 3.1.1. Off-Road Driving Assessment

The final off-road driving assessment consisted of five domains: basic information (five items), vehicle operation skills (18 items), physical function (10 items), sensory function (five items), and cognitive function (four items) (Table 2).

#### 3.1.2. On-Road Driving Assessment

The final on-road driving assessment consisted of 33 items reflecting real-world driving behaviors and situations, including pre-departure preparation, speed and directional control, road sign recognition, intersection management, maintenance of appropriate following distance, and responses to unexpected road situations. Each item was evaluated through direct observation and rated on a two-point scale (“Proficient” or “Not proficient”), with unassessable items marked as “Not applicable (N/A)” (Table 3).

### 3.2. Field Application of the Comprehensive Driving Evaluation

#### 3.2.1. General Characteristics of Participants

A total of 30 persons with disabilities participated in the field application of the comprehensive driving evaluation. The sample consisted of 15 males and 15 females. The largest age group was individuals in their 50s (n = 10, 33.3%). The most common disability type was severe cerebral palsy (n = 10, 33.3%), among whom two participants also presented with auditory impairment (Table 4).

#### 3.2.2. Driving Eligibility Outcomes

Based on the combined results of the comprehensive driving evaluation, three participants were classified as doubtful regarding fitness for independent driving. Overall driving eligibility outcomes for all participants, as determined independently by the driving instructor and the occupational therapist, are presented in Table 5.

Participant No. 8 (left hemiplegia due to moyamoya disease) demonstrated adequate cognitive test results; however, deficits in traffic situation recognition and appropriate response behaviors were observed. Sensory impairment and suspected unilateral neglect contributed to difficulty maintaining lane position, suggesting limitations in safe driving performance and raising concerns regarding fitness for independent driving.

Participant No. 23 showed cognitive impairment in the off-road assessment (TMT-B > 300 s with >3 errors). During the on-road driving assessment, the participant demonstrated difficulty maintaining forward attention and following instructions, including lane changes. Additional cognitive assessment was recommended. Participant No. 30 (severe auditory disorder) presented no apparent physical or cognitive impairments. However, limited auditory input and insufficient basic driving knowledge resulted in delayed responses to traffic situations. These findings indicated reduced readiness for safe independent driving.

## 4. Discussion

This study, conducted as part of a national initiative to strengthen the NRC’s driving rehabilitation system, demonstrates that the collaboratively developed CDE offers a structured and standardized alternative to the previously experience-based evaluation process. By integrating off-road functional assessment with an on-road driving test and clarifying the respective roles of occupational therapists and driving instructors, the CDE provides timely evidence to support the establishment of consistent operating procedures and professional competency requirements within Korea’s driving rehabilitation services.

Although the developmental process of this study may not represent the highest level of scientific rigor, it reflects the practical constraints of the Korean context, where the number of trained driving rehabilitation specialists and the available research evidence remain limited. Within these constraints, we prioritized feasibility and relevance, making full use of existing institutional resources and the operational environment of the National Rehabilitation Center to develop an assessment format that could be realistically implemented. By creating and applying a tool tailored to Korea’s cultural, regulatory, and service context, this study provides meaningful early evidence for countries where driving rehabilitation for persons with disabilities is still developing and offers a practical foundation for establishing context-appropriate evaluation procedures.

It should be noted that the term “comprehensive” in this study refers to the integration of off-road functional assessment with on-road driving evaluation, rather than to comprehensive coverage of all disability types. The field application was limited to persons with physical disabilities (primarily spinal cord injury and amputation) and cerebral palsy, which represent distinct evaluation challenges. The recent literature has increasingly emphasized the importance of diagnosis-specific assessment approaches, recognizing that evaluation demands differ substantially across conditions. For example, spinal cord injury primarily requires vehicle adaptation and operational assessment, whereas cerebral palsy involves complex interactions among motor, cognitive, visual, and perceptual impairments that necessitate a more in-depth clinical evaluation. Future research should develop and validate targeted assessment protocols tailored to specific diagnostic categories, building on the general framework established in this study.

The development of the off-road driving assessment was grounded in a systematic review of functional domains identified in the ICF Core Set for driving rehabilitation [12], which provided an essential framework for determining the key components required for safe driving. Building on this foundation, we incorporated elements from Jeong’s previously developed screening tool [20] and refined them through structured surveys and a workshop involving experienced occupational therapists. Throughout this process, we aimed to capture a comprehensive range of functions relevant to driving, including sensory, perceptual, cognitive, and motor domains [5,25]. Accordingly, we integrated standardized measures such as the Trail Making Test for cognitive flexibility and processing speed and the Clock Drawing Test for visual–perceptual and visual–spatial processing, and selected vision and motor assessments to ensure systematic and clinically meaningful coverage of the functional abilities required for on-road performance. This approach reflects our effort to construct an off-road evaluation that is both conceptually grounded and practically applicable within Korea’s emerging driving rehabilitation environment.

The necessity of including an on-road component in a comprehensive fitness-to-drive assessment is well established in the international literature. On-road assessments are widely regarded as the gold standard for evaluating driving fitness due to their ecological validity, as clinical off-road tests alone have been found to be insufficient to accurately differentiate between fit and unfit drivers [10,26]. This is particularly relevant for persons with disabilities, whose functional abilities in controlled clinical settings may not fully reflect their performance in real traffic environments.

During the early stages of this study, the research team visited Driving Mobility in the UK [26] to observe a working model that combines off-road and on-road assessment components. At the time, no comparable institutional model existed in Korea, and this visit represented our first opportunity to observe a structured collaborative assessment in an operational service setting. The Driving Mobility network employs both occupational therapists (or mobility clinicians) and approved driving instructors (termed “Driving Advisors”) who jointly conduct assessments across 20 centers in the UK. While this visit provided valuable practical insight into how combined assessment can be organized within a service delivery framework, we recognize that the UK model differs from the occupational therapy-led comprehensive driving evaluation systems more established in North America and Australia, where occupational therapists with specialized certification typically assume the primary role in fitness-to-drive determination [5,6]. Accordingly, future development of the Korean model should draw more broadly from international systems where the occupational therapist’s clinical role in outcome determination is more clearly defined. The field application in our study further supported this conclusion, demonstrating that the on-road test provided critical information for determining driving safety. Most members of the research team and NRC staff agreed on the necessity of including an on-road component in the Korean CDE.

We acknowledge that the international literature has raised concerns regarding the involvement of driving instructors in fitness-to-drive determination, noting that instructors may lack the medical knowledge needed to interpret why a driver with a specific condition makes particular errors during an on-road assessment. In more established systems, the occupational therapist typically leads the clinical decision-making process, while the driving instructor’s primary role is to ensure vehicle safety during the on-road evaluation and to provide operational training. In the present study, however, driving instructors were included in the determination process because they have historically been the sole evaluators within the Korean system, and the role of occupational therapists in driving rehabilitation has not yet been formally established. Notably, the three cases in which judgments differed between the two professionals (Participants No. 8, 23, and 30) illustrate the added value of occupational therapy assessment: in each case, the occupational therapist identified functional or cognitive concerns that were not detected through conventional instructor-based evaluation alone. These findings support the need for a clearer role delineation in future practice, in which the occupational therapist assumes primary responsibility for fitness-to-drive determination based on integrated clinical and on-road findings, while the driving instructor contributes operational expertise and ensures safety during assessment.

This collaborative model is particularly meaningful in Korea, where the role of occupational therapists in driving rehabilitation has not been formally established. Demonstrating effective interprofessional assessment provides a foundation for clarifying professional roles and aligns with international guidelines and certification frameworks such as ADED [5]. Moreover, the CDE model corresponds with national efforts to develop standardized training and evaluation pathways for disability-inclusive mobility services. As Korea expands community-based rehabilitation and strengthens mobility rights for persons with disabilities, structured driver assessment can offer essential evidence for policy advancement, including revision of driving eligibility regulations, formal integration of occupational therapists into driving rehabilitation systems, and the creation of reimbursement mechanisms for clinical driver evaluations.

This study has several limitations. First, as noted earlier, driving rehabilitation in Korea remains at an early stage of system development, with a limited number of trained specialists and a relatively small body of research evidence. The developmental process of this study was shaped by these practical constraints, prioritizing feasibility and relevance within the operational environment of the National Rehabilitation Center rather than pursuing the highest level of scientific rigor. Second, the pilot sample was modest in size and recruited from a single national rehabilitation center. Moreover, the sample was limited to persons with physical disabilities and cerebral palsy, and the assessment was not differentiated by diagnostic category. Future studies should include a broader range of conditions—such as traumatic brain injury, stroke, and neurodegenerative disorders—and examine whether diagnosis-specific modifications improve clinical accuracy. Multi-site implementation across regional driver rehabilitation programs is also needed to evaluate generalizability. Third, although expert review supported content validity, further psychometric analyses—including inter-rater reliability and predictive validity—are warranted. Finally, long-term follow-up assessing real-world driving outcomes after CDE clearance would enhance understanding of safety impact and clinical utility.

## 5. Conclusions

This study developed and applied a CDE that integrates structured off-road assessment with an on-road driving test through a collaborative occupational therapist–driving instructor model. The CDE proved feasible in real clinical settings and identified safety concerns that were not captured by experience-based judgment alone, demonstrating the value of combining functional testing with observed driving performance. Grounded in the ICF Core Set and refined through expert review, the assessment reflects both conceptual validity and practical relevance to Korea’s service environment. Although conducted within resource limitations, the study offers foundational evidence for establishing standardized driving evaluation procedures in Korea and supports future policy development and professional role expansion to improve mobility services for persons with disabilities.

## Figures and Tables

**Table 1:** Development and validation process of the comprehensive driving evaluation.

1	Development of the Comprehensive Driving Evaluation	Off-road driving assessment	Revision and supplementation of a previously reported assessment
			Expert advisory committee consultation
		On-road driving assessment	Literature review
			Preliminary draft development
			Survey of driving instructors
			Survey of the expert advisory committee
⇩			
2	Field Application of the Comprehensive Driving Evaluation	Telephone consultation	Coordination of the comprehensive driving evaluation schedule and location
		National Rehabilitation Center driving education program for persons with disabilities (driving instructor + occupational therapist)	Off-road driving assessment
			On-road driving assessment
			Preparation of the final comprehensive driving evaluation report

**Table 2:** Off-road driving assessment.

Section	Contents					
I. Basic Information	1. Participant Information					
	Diagnosis, Disability grade, Onset date, Cause of Disability, Mobility status, Assistive devices in use					
	2. Driving Information					
	Driver’s license, Driving experience, Vehicle ownership, Driving assistive devices in use					
	3. Medical Conditions					
	Ocular diseases, Cardiovascular diseases, Neurological disorders, Psychiatric disorders, Musculoskeletal disorders, Movement-related impairments					
	4. Medical History					
	5. Medications in Use					
II. Vehicle Operation Skills	Moving the vehicle	Operating the gear shift	Adjusting and checking the side mirrors		Operating the horn	Operating the parking brake
	Operating the headlights	Operating the turn signals	Adjusting and checking the rearview mirror		Steering wheel control	Operating the hazard lights
	Adjusting driving posture	Opening and closing the door	Fastening the seatbelt		Getting in and out of the driver’s seat	
	Adjusting the seat position	Operating the ignition switch	Operating the windshield wipers		Operating the accelerator and brake pedals	
III. Physical Function Assessment	Performance Ability (Able/Able with assistive device/Unable)					
	Checking both sides of the vehicle				Fastening the seatbelt	
	Operating the gear shift while steering				Operating the steering wheel	
	Moving between accelerator and brake pedals				Adjusting the seat position	
	Motor reaction speed				Adjusting driving posture	
	Gross motor movements					
IV. Sensory Function Assessment	Performance Ability (Able/Unable)					
	Visual acuity	Eye movement	Depth perception		Hearing	Proprioception
V. Cognitive Function Assessment	Trail Making Test—Part A			Trail Making Test—Part B		
	Clock Drawing Task			Snellgrove Maze Test		

**Table 3:** On-road driving assessment.

No.	Section	Assessment Item	Proficient	Not Proficient	N/A
1	Pre- Driving Preparation	Checks the surroundings of the vehicle for safety before departure.			
2		Checks the instrument panel before departure.			
3		Releases the parking brake before departure.			
4	Speed Control	Adjusts driving speed according to traffic conditions.			
5		Maintains the legal speed limit.			
6	Vehicle Operation	Controls the accelerator appropriately for the driving situation.			
7		Controls the brake appropriately for the driving situation.			
8		Changes gears appropriately for the driving situation.			
9		Holds and maneuvers the steering wheel smoothly.			
10	Driving	Monitors the surrounding environment through mirrors while driving.			
11	Vehicle Awareness	Maintains an appropriate distance from other vehicles.			
12		Keeps the vehicle within the lane without deviation.			
13	Lane Change	Maintains a safe distance when changing lanes.			
14		Uses turn signals appropriately when changing lanes.			
15		Actively checks for safety by turning head or using mirrors when changing lanes.			
16	Stopping	Stops smoothly without abrupt braking.			
17		Makes a complete stop at the stop line.			
18	Traffic Signals	Identifies traffic signals and responds promptly.			
19		Anticipates upcoming situations based on signal changes.			
20	Road Signs	Understands and follows the meaning of road signs.			
21	Unprotected Left Turn	Makes an unprotected left turn with adequate clearance from oncoming vehicles.			
22	Traffic Awareness	Continuously checks mirrors to monitor traffic conditions.			
23	Intersection & Roundabout	Yields the right of way appropriately.			
24		Approaches intersections with sufficient caution and distance.			
25	Safety Awareness	Recognizes and yields to pedestrians.			
26		Checks blind spots through mirrors.			
27		Responds appropriately to unexpected situations.			
28	Driving Behavior	Drives calmly without panic or excessive tension.			
29	Attention	Maintains concentration and responds to driving stimuli.			
30		Pays attention to surrounding traffic when overtaking.			
31		Monitors other vehicles carefully.			
32		Follows the designated route and arrives at the destination accurately.			
33	Parking	Identifies parking space and location, parks accurately and safely at an appropriate speed.			

**Table 4:** General characteristics of participants (n = 30).

Section		n	%
Gender	Male	15	50.0
	Female	15	50.0
Age (years)	20–29	4	13.3
	30–39	3	10.0
	40–49	6	20.0
	50–59	10	33.3
	60–69	7	23.4
Region	Capital area (Seoul, Gyeonggi, Incheon)	17	56.7
	Yeongnam area (Daegu, Busan, Ulsan)	10	33.3
	Chungcheong area (Daejeon, Chungbuk)	2	6.7
	Gangwon area (Donghae)	1	3.3
	Honam area	0	0.0
Type of Disability	Cerebral palsy (severe)	10	33.3
	Physical disability (mild)	7	23.4
	Physical disability (severe)	7	23.3
	Cerebral palsy (mild)	4	13.3
	Auditory disorder (severe)	2	6.7

**Table 5:** Driving eligibility results for all participants.

No.	Gender	Age	Type of Disability	Diagnosis	Driving Eligibility	
					Driving Instructor	Occupational Therapist
1	F	49	Cerebral Palsy (severe)	Right Hemiplegia	Eligible	Eligible
2	M	45	Cerebral Palsy (severe)	Right Hemiplegia	Eligible	Eligible
3	F	29	Cerebral Palsy (severe)	Left Hemiplegia	Eligible	Eligible
4	M	33	Cerebral Palsy (severe)	Right Hemiplegia	Eligible	Eligible
5	M	62	Cerebral Palsy (severe)	Left Hemiplegia	Eligible	Eligible
6	M	65	Cerebral Palsy (severe)	Left Hemiplegia	Eligible	Eligible
7	M	57	Cerebral Palsy (severe)	Right Hemiplegia	Eligible	Eligible
8	F	33	Cerebral Palsy (severe)	Left Hemiplegia	Eligible	Doubtful *
9	F	50	Cerebral Palsy (severe)	Right Hemiplegia	Eligible	Eligible
10	F	56	Cerebral Palsy (severe)	Right Hemiplegia	Eligible	Eligible
11	F	63	Physical Disability (severe)	Poliomyelitis	Eligible	Eligible
12	F	63	Physical Disability (severe)	Spinal Cord Injury (T12)	Eligible	Eligible
13	M	52	Physical Disability (severe)	Spinal Cord Injury (T5–7)	Eligible	Eligible
14	M	54	Physical Disability (severe)	Spinal Cord Injury (T5–6)	Eligible	Eligible
15	M	63	Physical Disability (severe)	Spinal Cord Injury (T5)	Eligible	Eligible
16	M	20	Physical Disability (severe)	Spinal Cord Injury (T5–6)	Eligible	Eligible
17	M	49	Physical Disability (severe)	Bilateral Ankle Amputation	Eligible	Eligible
18	M	49	Physical Disability (mild)	Spinal Cord Disorder (T3–4)	Eligible	Eligible
19	F	56	Physical Disability (mild)	Right Knee Amputation	Eligible	Eligible
20	F	51	Physical Disability (mild)	Right Lower Limb Amputation	Eligible	Eligible
21	F	62	Physical Disability (mild)	Poliomyelitis	Eligible	Eligible
22	F	57	Physical Disability (mild)	Poliomyelitis	Eligible	Eligible
23	F	55	Physical Disability (mild)	Congenital Spinal Defect	Eligible	Doubtful *
24	M	59	Physical Disability (mild)	Joint Disorder (Right Knee)	Eligible	Eligible
25	M	29	Cerebral Palsy (mild)	Right Hemiplegia	Eligible	Eligible
26	M	36	Cerebral Palsy (mild)	Left Hemiplegia	Eligible	Eligible
27	F	42	Cerebral Palsy (mild)	Right Hemiplegia	Eligible	Eligible
28	M	64	Cerebral Palsy (mild)	Right Hemiplegia	Eligible	Eligible
29	F	23	Auditory Disorder (severe)	Auditory Disorder	Eligible	Eligible
30	F	48	Auditory Disorder (severe)	Auditory Disorder	Eligible	Doubtful *

* Indicates cases in which driving eligibility judgments differed between the driving instructor and the occupational therapist.

## Data Availability

The data presented in this study are managed by the National Rehabilitation Center and are not publicly available due to institutional data governance and ethical restrictions.

## Abbreviations

The following abbreviations are used in this manuscript:

CDE	Comprehensive driving evaluation
NRC	National Rehabilitation Center
ICF	International Classification of Functioning, Disability and Health
ORDA	Off-road driving assessment
TMT-A	Trail Making Test—Part A
TMT-B	Trail Making Test—Part B
